# Characterizing the Inflammatory Microenvironment in K14-HPV16 Transgenic Mice: Mast Cell Infiltration and MicroRNA Expression

**DOI:** 10.3390/cancers14092216

**Published:** 2022-04-28

**Authors:** Alexandra C. Costa, Joana M. O. Santos, Beatriz Medeiros-Fonseca, Paula A. Oliveira, Margarida M. S. M. Bastos, Haissa O. Brito, Rui M. Gil da Costa, Rui Medeiros

**Affiliations:** 1 Molecular Oncology and Viral Pathology Group, Research Center of IPO Porto (CI-IPOP)/RISE@CI-IPOP (Health Research Network), Portuguese Oncology Institute of Porto (IPO Porto), Porto Comprehensive Cancer Center (Porto.CCC), 4200-072 Porto, Portugal; alexandracastrocosta@gmail.com (A.C.C.); joana.oliveira.santos@ipoporto.min-saude.pt (J.M.O.S.); rmcosta@fe.up.pt (R.M.G.d.C.); 2 Faculty of Medicine of the University of Porto (FMUP), 4200-319 Porto, Portugal; 3 Research Department of the Portuguese League against Cancer—Regional Nucleus of the North (Liga Portuguesa Contra o Cancro—Núcleo Regional do Norte), 4200-177 Porto, Portugal; 4 Centre for the Research and Technology of Agro-Environmental and Biological Sciences (CITAB), Inov4Agro, University of Trás-os-Montes e Alto Douro (UTAD), Quinta de Prados, 5000-801 Vila Real, Portugal; fonsecabeatriz@live.com.pt (B.M.-F.); pamo@utad.pt (P.A.O.); 5 LEPABE—Laboratory for Process Engineering, Environment, Biotechnology and Energy, Faculty of Engineering, University of Porto, Rua Dr. Roberto Frias, 4200-465 Porto, Portugal; mbastos@fe.up.pt; 6 ALiCE—Associate Laboratory in Chemical Engineering, Faculty of Engineering, University of Porto, Rua Dr. Roberto Frias, 4200-465 Porto, Portugal; 7 Postgraduate Programme in Adult Health (PPGSAD), Department of Morphology, Federal University of Maranhão (UFMA), and UFMA University Hospital (HUUFMA), São Luís 65080-805, Brazil; haissa.brito@ufma.br; 8 Virology Service, Portuguese Oncology Institute of Porto (IPO Porto), 4200-072 Porto, Portugal; 9 Biomedical Research Center (CEBIMED), Faculty of Health Sciences of the Fernando Pessoa University, 4249-004 Porto, Portugal

**Keywords:** HPV16, inflammation, mast cells, K14-HPV16, microRNAs, cancer, carcinogenesis

## Abstract

**Simple Summary:**

K14-HPV16 transgenic mice have proved to be a useful model to study the carcinogenic cascade induced by HPV16, the tumor microenvironment and also the epigenetic and genetic factors associated with this type of malignancy. The aim of our study was to evaluate the infiltration of mast cells in two cutaneous regions with different severity of the lesions and to identify potential microRNAs that may regulate mast cell infiltration in this model. We were able to confirm that increased mast cell infiltration is associated with progression of HPV-induced lesions, and that miR-223-3p and miR-125b-5p might be assisting this process via the regulation of mast cell chemotactic proteins.

**Abstract:**

High-risk human papillomavirus (HPV) is the etiologic agent of several types of cancer. Mast cells’ role as either a driving or opposing force for cancer progression remains controversial. MicroRNAs are dysregulated in several HPV-induced cancers, and can influence mast cell biology. The aim of this study was to evaluate mast cell infiltration and to identify microRNAs potentially regulating this process. Transgenic male mice (K14-HPV16; HPV^+^) and matched wild-type mice (HPV^−^) received 7,12-Dimethylbenz[a]anthracene (DMBA) (or vehicle) over 17 weeks. Following euthanasia, chest skin and ear tissue samples were collected. Mast cell infiltration was evaluated by immunohistochemistry. MicroRNAs associated with mast cell infiltration were identified using bioinformatic tools. MicroRNA and mRNA relative expression was evaluated by RT-qPCR. Immunohistochemistry showed increased mast cell infiltration in HPV^+^ mice (*p* < 0.001). DMBA did not have any statistically significant influence on this distribution. Ear tissue of HPV^+^ mice showed increased mast cell infiltration (*p* < 0.01) when compared with chest skin samples. Additionally, reduced relative expression of miR-125b-5p (*p* = 0.008, 2^−ΔΔCt^ = 2.09) and miR-223-3p (*p* = 0.013, 2^−ΔΔCt^ = 4.42) seems to be associated with mast cell infiltration and increased expression of target gene *Cxcl10*. These results indicate that HPV16 may increase mast cell infiltration by down-regulating miR-223-3p and miR-125b-5p.

## 1. Introduction

High-risk human papillomavirus (HPV) is responsible for cancers in several anatomic regions, including the cervix, vulva, vagina, anus, penis, and oropharynx [1]. HPV16 and HPV18 are the high-risk type most commonly found in most of those cancers. In high-risk HPV, the E6, E7 and E5 proteins play an oncogenic role due to their capacity to form specific complexes with tumor suppressor proteins [2]. Importantly, these oncoproteins have the capacity to induce pro-inflammatory signaling pathways [3,4]. Such pro-inflammatory stimuli, along with chronic inflammation resulting from host antiviral response to persistent HPV infection, are essential for tumor development [3,4]. The inflammatory tumor microenvironment is composed by pre-malignant and malignant cells, stromal cells, and innate and adaptative immune cells [5], which interact with each other, modulating cancer development [5]. Mast cells influence other cells, both through soluble mediators and cell-to-cell interactions [6]. The role of mast cells in cancer progression is ambiguous, since they can promote an anti-tumor response or contribute to tumor growth [7,8]. The pro-tumoral functions of mast cells include the promotion of metastasis, the release of pro-angiogenic factors and the participation in immunosuppression and cancer-related inflammation [9]. Additionally, mast cell accumulation has been associated with poor prognosis and reduced survival in several solid tumors [10]. The role of mast cells in high-risk HPV-induced carcinogenesis and the mechanisms that regulate their accumulation in the tumor microenvironment are poorly defined.

MicroRNAs (miRNAs/miRs) are small and conserved RNA molecules that play an important role in the regulation of gene expression [11]. The oncoproteins of high-risk HPV are able to alter the expression of cellular miRNAs, contributing to malignant transformation [11]. Further, miRNAs are key players in modulating the tumor microenvironment, including the infiltration and activity of immune cells [12,13]. However, there is not much information regarding the effects of miRNAs in mast cells in the context of cancer development. The HPV oncoproteins may deregulate miRNAs that control mast cell migration, modulating their infiltration into the tumor microenvironment. Therefore, in this study, we sought to evaluate the infiltration of mast cells into HPV16-driven lesions in transgenic mice carrying all of the HPV16 early genes [14,15]. Additionally, we aimed to identify potential microRNAs that may regulate mast cell infiltration in this model.

## 2. Materials and Methods

### 2.1. Animals

Generation of K14-HPV16 mice on an FVB/n background has been previously reported [16]. These transgenic mice were kindly donated by Dr. Jeffrey Arbeit and Dr. Douglas Hanahan (University of California) through the USA National Cancer Institute Mouse Repository. The animal experiments were approved by the University of Trás-os-Montes and Alto Douro Ethics Committee (approval no. 10/2013) and the Portuguese General Veterinary Directorate (0421/000/000/2014). Animals were housed and bred according to Portuguese (Decreto-Lei 113, 7 August) and European (EU Directive 2010/63/EU) legislation, under controlled temperature (23 ± 2 °C), light-dark cycle (12 h light/12 h dark) and relative humidity (50 ± 10%). Food and water were provided ad libitum.

### 2.2. Experimental Design and Sample Collection

The chest and ear samples and experimental design were previously used by Peixoto da Silva et. al., 2020 [17]. Briefly, 34 male mice were randomly allocated in four groups: Group 1 (*n* = 9, HPV^−^ mice, without 7,12-Dimethylbenz[a]anthracene (DMBA)), Group 2 (*n* = 5, HPV^−^ mice, with DMBA), Group 3 (*n* = 10, HPV^+^ mice, without DMBA) and Group 4 (*n* = 10, HPV^+^ mice, with DMBA). The DMBA (D3254, Sigma-Aldrich, Merck kGAa, Darmstadt, Germany) was dissolved in dimethyl sulfoxide (DMSO) (CARLO ERBA Reagents S.A.S., Val de Reuil, France) and topically administered to the penile mucosa once a week (0.031 mg/animal/week in 4 μL of DMSO), starting at 9–11 weeks during 17 weeks of the experiment. All of the mice were sacrificed at 31–33 weeks of age under ketamine (CLORKETAM 1000, injectable solution, Vétoquinol, Barcarena, Portugal) and xylazine (Rompun^®^ 2%, Bayer Healthcare S.A., Kiel, Germany) anesthesia, by intracardiac puncture and exsanguination, as indicated by the Federation for Laboratory Animal Science Associations (FELASA). The chest skin and ear tissue samples were weighed and collected into TripleXtractor reagent (Grisp^®^, Porto, Portugal), macerated and kept at −80 °C until further use. Matched chest skin and ear samples were also collected for histological analysis (histological results previously published by [17]).

### 2.3. Immunohistochemistry

Formalin-fixed, paraffin-embedded (FFPE) tissue sections were fixed, deparaffinized in xylene, rehydrated in a graded series of alcohol, and subjected to antigen retrieval through citrate buffer and microwave heating for 15 min and then cooled for 20 min at room temperature. The slides were covered for 5 min with peroxidase blocking reagent (Novolink Polymer Detection System, Novocastra, Leica Biosystems, Germany), followed by washing with PBS 1×-Tween 0.05% (PBS-T), and protein block (Novolink Polymer Detection System, Novocastra) was used for 5 min. Individual slides were incubated for 1 h 15 min with anti-TPSAB-1 (SC68-07; ThermoFisher) antibody diluted in PBS 1× (1:200 dilution), at room temperature. The slides were then washed with PBS-T and incubated with Novolink Polymer (30 min) and washed with PBS-T again. The color reaction was developed in DAB Chromogen (Novolink Polymer Detection System, Novocastra) according to the manufacturer’s instructions. The sections were then counterstained with hematoxylin, dehydrated and mounted. Positive controls consisted of sections of lymph node tissues, while for negative controls the primary antibody was omitted (Figure S1). The staining was assessed and validated by an experienced pathologist (RMGC). To evaluate mast cell infiltration, the number of immunostained cells was determined in at least five high magnification fields (500×) for each animal, randomly chosen from the superficial dermis, representing the dermo-epidermal interface, and counting was performed in ImageJ. The results were presented as the mean and standard deviation of the animals in each group.

### 2.4. MicroRNA Selection

To retrieve the proteins associated with mast cell infiltration we created a protein–protein interaction (PPI) network using Cytoscape 3.7.2. The Search Tool for the Retrieval of Interacting Genes/Proteins (STRINGapp 1.5.1), installed on Cytoscape, was used to search and visualize the proteins associated with mast cell infiltration and mast cell chemotaxis through Pubmed Query. A confidence (score) cutoff of 0.4 and a number of proteins of 100 were selected. The PPI network was further clustered using Clustermaker 2 v.1.3.1, to identify densely connected regions and clusters of proteins that were crucial in the PPI network. The CytoHubba (version 0.1) plugin Cytoscape was also utilized to identify the key proteins within the higher cluster, according to maximal clique centrality (MCC). Finally, for the top 10 proteins, we identified microRNAs that regulate their respective messenger RNAs, using miRNA databases (TargetScan, miRDB, miRmap, miRwalk, miRTarBase and TarBase) [18,19,20,21,22,23]. The four microRNAs with higher scores in those databases and related to the majority of the genes in our network were selected.

### 2.5. Total RNA Isolation

Total RNA extraction from chest skin and ear samples was performed using TripleXtractor reagent (Grisp^®^, Portugal) followed by a chloroform solution (EMSURE^®^, Merck kGAa, Darmstadt, Germany). Total RNA was then purified using a commercial kit, GRS total RNA kit (Grisp^®^, Portugal), according to the manufacturer’s instructions. DNAse treatment was included for all of the samples. RNA concentration and purity were measured using the NanoDrop Lite spectrophotometer (Thermo Scientific^®^). 

### 2.6. cDNA Synthesis

The microRNA samples (50 ng) were used as templates for cDNA synthesis using the TaqMan^®^ MicroRNA Reverse Transcription Kit (Applied Biosystems^®^) and sequence-specific stem-loop reverse transcription primers for miR-223-3p, miR-466l-3p, miR-466k, miR-125b-5p and snoRNA-202. The conversion was performed in a Biometra^®^ Personal Cycler (Biocompare, USA) with the following conditions: 30 min at 16 °C, 60 min at 42 °C and 10 min at 85 °C. Total RNA samples (300 ng) were also used as templates for cDNA synthesis using the High-Capacity cDNA Reverse Transcription Kit (Applied BiosystemTM). The conversion was performed in a Biometra^®^ Personal Cycler (Biocompare, USA) with the following conditions: 10 min at 25 °C, 120 min at 37 °C, and 5 min at 85 °C. Negative controls lacking RNA were also included in all reactions.

### 2.7. Relative Quantification of MicroRNAs

Each reaction was performed with 5 µL of 2× TaqMan^®^ Fast Advanced Master Mix (Applied Biosystems^®^) with 0.5 µL of 20× TaqMan^®^ MicroRNA Expression Assays (miR-466l-3p: 002804; miR-466k: 240990_mat; miR-223-3p: 002295; miR-125b-5p: 000449; snoRNA-202: 001232; Applied Biosystems^®^), 3 µL of nuclease-free water and 1.5 µL of cDNA sample, making a total volume of 10 µL. The quantification was performed in duplicate, and CT standard deviation values superior to 0.5 were excluded. Negative controls lacking cDNA were also included in all reactions. All target and endogenous controls for each sample were amplified in the same plate. The thermal cycling conditions for all assays were the following: 20 s at 95 °C followed by 45 cycles of 1 s at 95 °C and 20 s at 60 °C. The same baseline and threshold were set for each plate using the analysis software for qPCR from the Thermo Fisher Connect platform (Thermo Fisher Scientific, Waltham, MA, USA), in order to generate threshold cycle (Ct) values for all of the miRs/SnoR in each sample. Small nucleolar RNA 202 (snoR-202) was previously tested by our group in this mouse model, was the one that showed the lowest standard deviation values in chest and ear, and therefore, was used as endogenous control [14,15].

### 2.8. Relative Quantification of MRNAs

Each reaction was performed with 5 µL of 2× TaqMan^®^ Fast Advanced Master Mix (Applied Biosystems^®^) with 0.5 µL of 20× TaqMan^®^ Gene Expression Assays (Ccr2: Mm00438270_m1; Cxcl10: Mm00445235_m1; β-actin: Mm01205647_g1; Applied Biosystems^®^), 3.5 µL of nuclease-free water and 1 µL of cDNA sample, making a total volume of 10 µL. The quantification was performed in duplicate, and CT standard deviation values superior to 0.5 were excluded. Negative controls lacking cDNA were also included in all reactions. All target and endogenous controls for each sample were amplified in the same plate. The thermal cycling conditions for all assays were the following: 20 s at 95 °C followed by 45 cycles of 1 s at 95 °C and 20 s at 60 °C. The same baseline and threshold were set for each plate using the analysis software for qPCR from the Thermo Fisher Connect platform (Thermo Fisher Scientific, Waltham, MA, USA), in order to generate Ct values for all of the genes in each sample. β-actin was previously tested by our group in chest skin and ear tissue samples of this mice model, was the one that showed the lowest standard deviation values, and therefore, was used as endogenous control.

### 2.9. Statistical Analysis

Statistical analysis was performed using IBM SPSS Statistics for Windows (Version 27.0). Immune cell infiltration was evaluated using the Mann–Whitney *U* test and the Kruskal–Wallis test. Additionally, to analyze mast cell distribution in the different histological lesions, a Fisher–Freeman–Halton test was performed. In order to understand which microRNAs were influencing the various degrees of mast cell infiltration, we used the Kruskal–Wallis test. Finally, the presence of statistical differences in microRNA expression was evaluated using the Livak method along with Mann–Whitney *U* test. All of the graphics were constructed using GraphPad Prism 8 (GraphPad Software). The results were considered statistically significant when the *p* values were <0.05.

## 3. Results

### 3.1. Mast Cell Infiltration 

**Figure 1:**
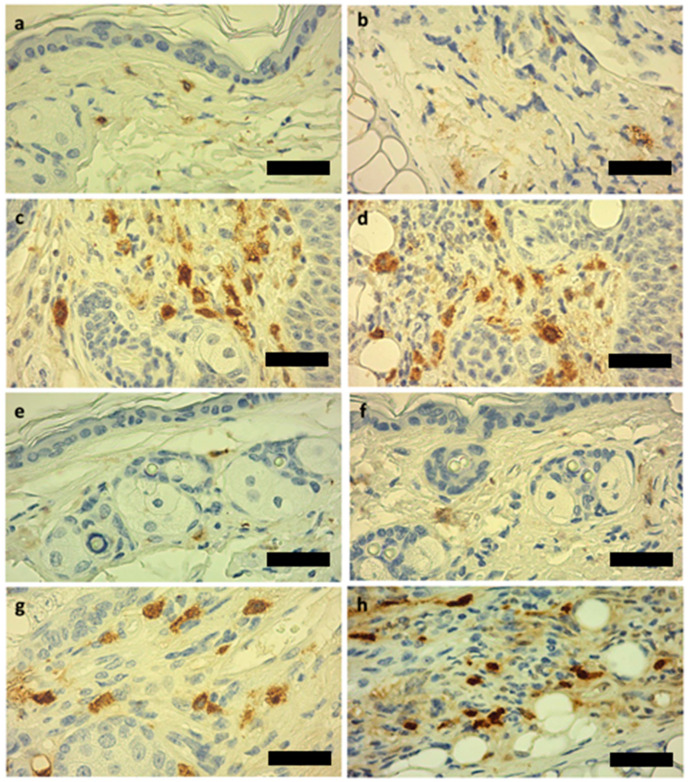
Histological analysis of mice chest skin and ear tissue samples, 500×, DAB-hematoxylin, scale bar = 20 µm. (**a**) Chest skin with low mast cell infiltration observed in wild-type (HPV^−^) mice. (**b**) Normal ear with low mast cell infiltration observed in HPV^−^ mice. (**c**) Mast cell infiltration observed in chest skin of K14-HPV16 (HPV^+^) transgenic mice. (**d**) Mast cell infiltration observed in ear tissue of HPV^+^ mice. (**e**) Chest skin of an HPV^−^ mouse with DMBA treatment. (**f**) Ear tissue of an HPV^−^ mouse with DMBA treatment. (**g**) Chest skin of HPV^+^ transgenic mouse with DMBA presence. (**h**) Ear tissue of HPV^+^ transgenic mouse with DMBA presence.

**Figure 2:**
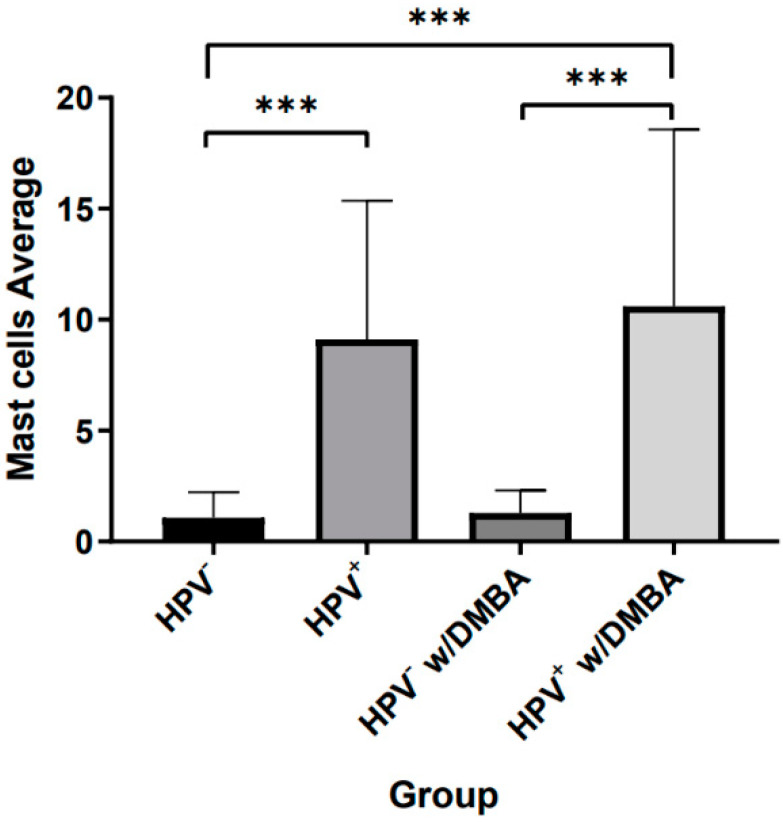
Mast cell infiltration in chest skin and ear tissue in the different groups. Transgenic mice (HPV^+^) had a higher infiltration of mast cells when compared to the wild-type (HPV^−^) (*** *p* < 0.001). DMBA presence did not alter the predisposition for mast cell infiltration in any of the groups.

To determine if HPV was able to modulate mast cell infiltration in both chest skin and ear tissues of the K14-HPV16 transgenic mice, we evaluated the infiltration of mast cells in FFPE tissue specimens by immunohistochemistry using a specific anti-mast cell tryptase antibody (TPSAB-1). We observed that, in general, mast cell infiltration increased substantially in the transgenic mice (HPV^+^) when compared with the wild type (HPV) (*p* < 0.001) (Figure 1 and Figure 2). The presence or absence of DMBA did not have any statistically significant influence on this distribution (*p* = 0.650 in wild-type mice and *p* = 0.568 in transgenic mice) (Figure 2).

**Figure 3:**
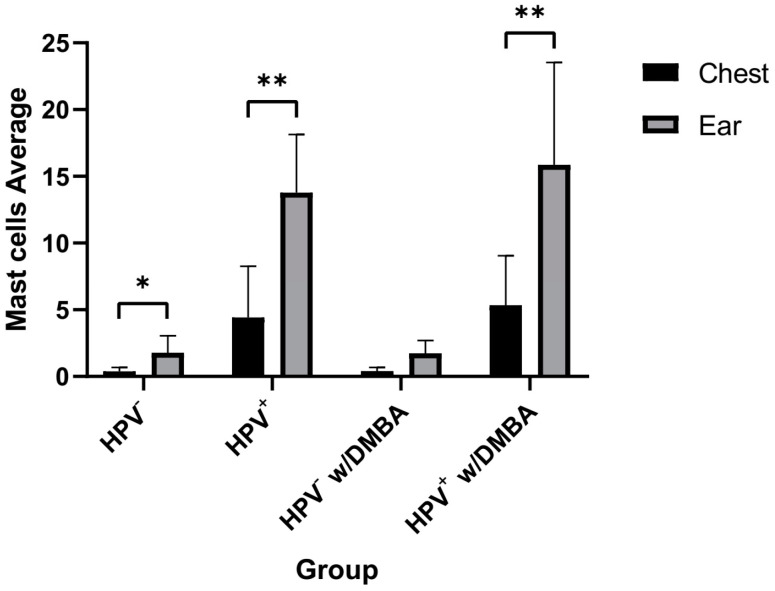
Mast cell differential infiltration in each group (chest skin and ear tissue together). There was an increase in mast cell infiltration in the ear tissue when compared to the chest skin, in both wild-type and transgenic mice. HPV^−^ had a statistically lower difference between both organs (* *p* < 0.05), whereas in HPV^+^ without and with DMBA, there was a higher statistically significant difference (** *p* < 0.01).

We also evaluated mast cell infiltration in the two organs separately. The mean number of mast cells was higher in ear tissue when compared with chest skin (Figure 3). This alteration was perceptible in both wild-type (HPV^−^) and transgenic mice (HPV^+^), although it was much more evident in the HPV^+^ groups, both without and with DMBA (*p* < 0.01) (Figure 3).

**Figure 4:**
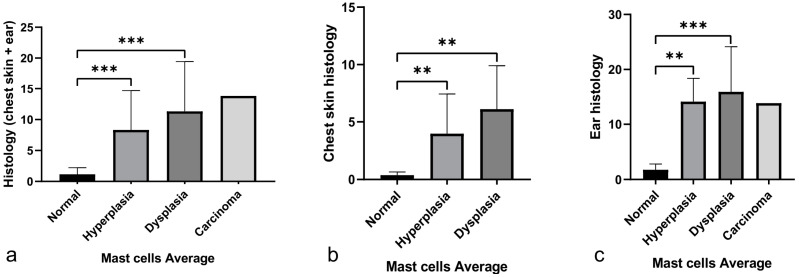
Mast cell infiltration in lesion progression in both organs together (**a**), chest skin (**b**) and ear tissue (**c**). (**a**) Statistically significant differences were found when comparing the normal epithelium versus the hyperplastic epithelium (*** *p* < 0.001) and between the normal epithelium versus the dysplastic epithelium (*** *p* < 0.001). (**b**) In chest skin, comparing the normal epithelium with the hyperplastic and dysplastic epithelium, we observed an increase in mast cell infiltration (** *p* < 0.01). (**c**) In ear tissue, we also found statistically significant differences between the normal and hyperplastic epithelium (** *p* < 0.01) and between the normal and dysplastic epithelium (*** *p* < 0.001).

Next, we wanted to understand if mast cell infiltration increased as lesions progressed from normal epidermis to epidermal hyperplasia, dysplasia and squamous cell carcinoma. Analyzing both organs together, we observed important differences between the normal epithelium and the hyperplastic epithelium (*p* < 0.001), as well as between the normal epithelium and the dysplastic epithelium (*p* < 0.001) (Figure 4a). No statistically significant difference was observed between the hyperplastic epithelium and the dysplastic epithelium (*p* = 0.348) (Figure 4a). The evaluation of chest skin alone showed, once again, differences between the normal epithelium when compared with both hyperplastic and dysplastic epithelium (*p* = 0.04 and *p* = 0.03, respectively) (Figure 4b). The same occurred when we analyzed only the ear tissue (*p* < 0.001) (Figure 4c). The single carcinoma sample could not be analyzed statistically, but mast cell infiltration was detected and mast cell counts were similar to those from dysplastic lesions (Figure 4c).

In order to determine how mast cells were distributed in the different histological lesions, we ranked mast cell averages in terciles, using the IBM SPSS software platform. A rank of 1 was assigned to a mean of 0 to 2 mast cells per camp and represented a low mast cell count; a rank of 2 was assigned to a mean of 2 to 9 mast cells per camp and represented an intermediate mast cell count; and finally, a rank of 3 was assigned to a mean of 9 to 33 mast cells per camp and represented a high mast cell count. A low mast cell count was mainly observed in normal samples, an intermediate mast cell count was associated with 4 hyperplastic samples and 8 dysplastic samples, and the highest mast cell count was found homogeneously distributed between the hyperplastic and dysplastic lesions (*p* < 0.001) (Table 1a). By analyzing each organ separately, we were able to confirm that more advanced lesions had an increase in the mast cell count (*p* = 0.002 and *p* < 0.001 for Table 1b,c respectively).

### 3.2. Identification of Proteins and MicroRNAs Potentially Involved in Mast Cell Infiltration 

**Figure 5:**
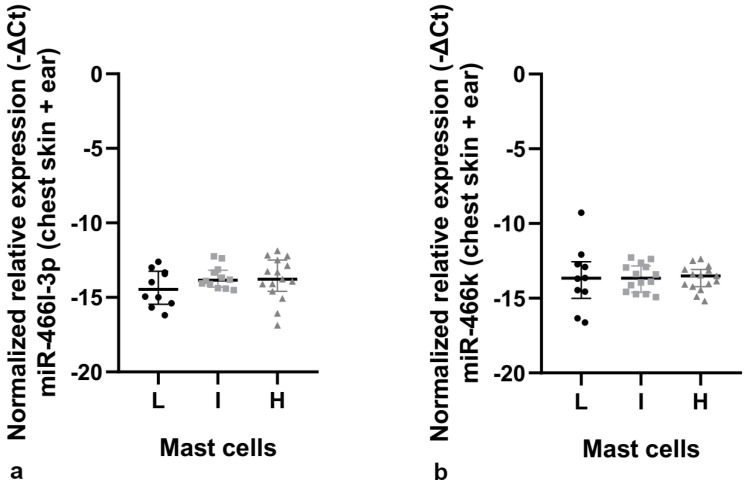
MiR-466l-3p and MiR-466k relative expression in K14-HPV16 transgenic mice. (**a**) MiR-466l-3p relative expression in chest skin and ear tissue together. (**b**) MiR-466k relative expression in chest skin and ear tissue together. L: low mast cell count; I: intermediate mast cell count; H: high mast cell count.

To identify proteins related with mast cell infiltration, we used StringApp 1.6.0 in Cytoscape 3.7.2. The top 100 proteins were retrieved with a confidence cut-off of 0.4. Next, using clusterMaker2, we applied the Markov clustering (MCL) to promote the clustering. We obtained clusters of proteins that had more interaction among themselves than with the rest of the network. Then, for the major cluster obtained, the Cytohubba 0.1 app was used to identify the top 10 hub proteins (Table 2). Having found the essential proteins associated with mast cell infiltration (Table 2), we then searched in several miRNA online databases for miRNAs that target the messenger RNA of those top 10 proteins and retrieved the four microRNAs with higher scores, namely miR-466l-3p, miR-466k, miR-223-3p and miR-125b-5p (Table 3). Next, we tried to understand if these four microRNAs could be associated with mast cell infiltration in chest skin and ear tissue of K14-HPV16 transgenic mice. Performing a Kruskal–Wallis test between the relative expression of the four microRNAs and the terciles obtained previously (low, intermediate and high mast cell count), we found that only miR-223-3p and miR-125b-5p had statistically significant results (*p* = 0.035 and *p* = 0.024, respectively), meaning that only these two microRNAs were influencing mast cell infiltration (Table 4). For miR-466l-3p and miR-466k, no statistical differences were found, so no further evaluation was performed (Table 4 and Figure 5).

### 3.3. MiR-223-3p Is Associated with Mast Cell Infiltration

**Figure 6:**
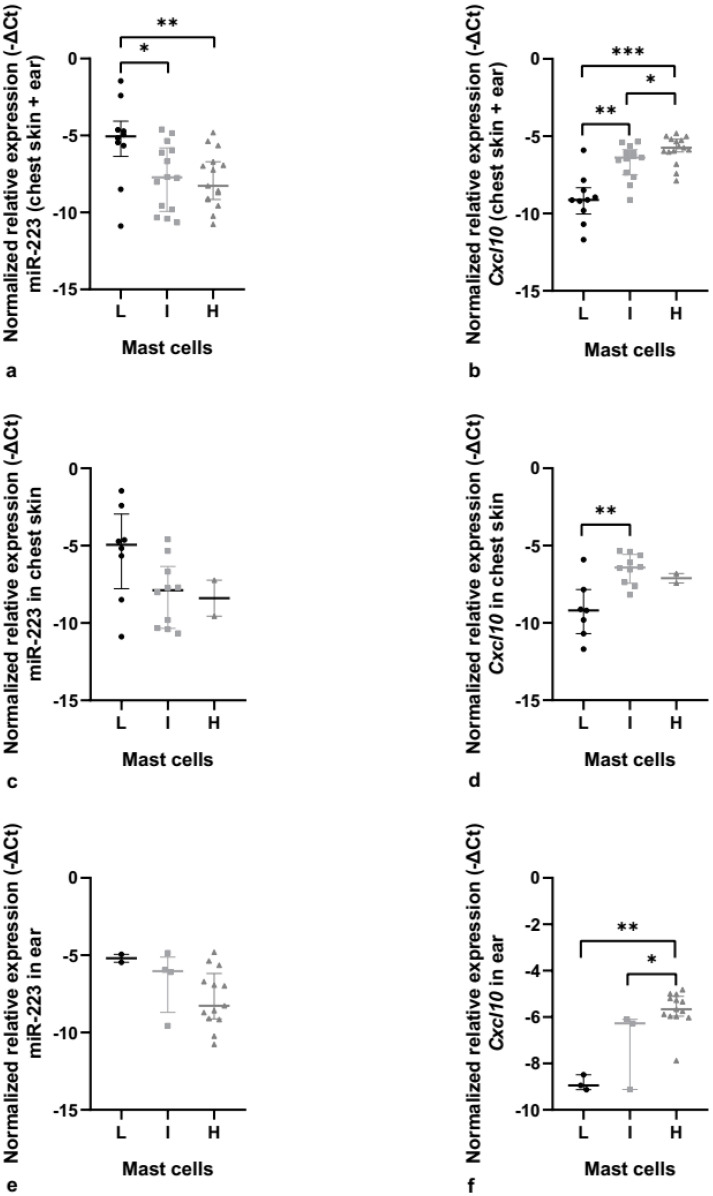
MiR-223 and *Cxcl10* relative expression in K14-HPV16 transgenic mice. (**a**) MiR-223 relative expression in chest skin and ear tissue, together. There are statistically significant differences between the low and intermediate mast cell counts (* *p* < 0.05) and between the low and high mast cell counts (** *p* < 0.01). (**b**) *Cxcl10* relative expression in chest skin and ear tissue, together. We observed statistically significant results between the low and intermediate mast cell counts (** *p* < 0.01), between the low and high mast cell counts (*** *p* < 0.001), and between the intermediate and high mast cell counts (* *p* < 0.05). (**c**) Normalized relative expression of miR-223 in chest skin. (**d**) Normalized relative expression of *Cxcl10* in chest skin. Statistically significant differences were found between the low and intermediate mast cell counts (** *p* < 0.01). (**e**) MiR-223 relative expression in ear tissue. (**f**) *Cxcl10* relative expression in ear tissue. Statistically significant results were observed between the low and high mast cell counts (** *p* < 0.01) and between the intermediate and high mast cell counts (* *p* < 0.05). L: low mast cell count; I: intermediate mast cell count; H: high mast cell count.

To confirm our hypothesis that miR-223-3p could be associated with mast cell infiltration in this transgenic mouse model, we first conducted a joint analysis of both organs, and observed that there were statistically significant differences between the low mast cell count and the high mast cell count (*p* = 0.013; 2^−ΔΔCt^ = 4.42) and also between the low mast cell count and the intermediate mast cell count (*p* = 0.040; 2^−ΔΔCt^ = 4.85) (Figure 6a). 

Since Cxcl10 is one of the essential proteins that is targeted by miR-223-3p and mast cell infiltration, we evaluated its expression and observed that there were statistically significant differences between the low mast cell count and the intermediate and high mast cell counts (*p* = 0.002; 2^−ΔΔCt^ = 5.37 and *p* < 0.001; 2^−ΔΔCt^ = 9.30, respectively) and also between the intermediate mast cell count and the high mast cell count (*p* = 0.023; 2^−ΔΔCt^ = 1.73) (Figure 6b).

When we performed a more specific analysis and centered our attention on chest skin, we did not find any statistically significant difference when analyzing the miR-223 relative expression (Figure 6c,e). Nonetheless, when analyzing *Cxcl10* expression, we observed a statistically significant difference between the low mast cell count and the intermediate mast cell count (*p* = 0.008; 2^−ΔΔCt^ = 6.38) (Figure 6d). The same happened when we analyzed the ear tissue separately, where miR-223 did not have any statistically significant differences (Figure 6e), but when we focused on *Cxcl10* expression, we observed statistically significant differences between the low mast cell count and high mast cell count (*p* = 0.009; 2^−ΔΔCt^ = 9.08) and also between the intermediate mast cell count and high mast cell count (*p* = 0.019; 2^−ΔΔCt^ = 2.81) (Figure 6f).

### 3.4. MiR-125b-5p Is Associated with Mast Cell Infiltration

**Figure 7:**
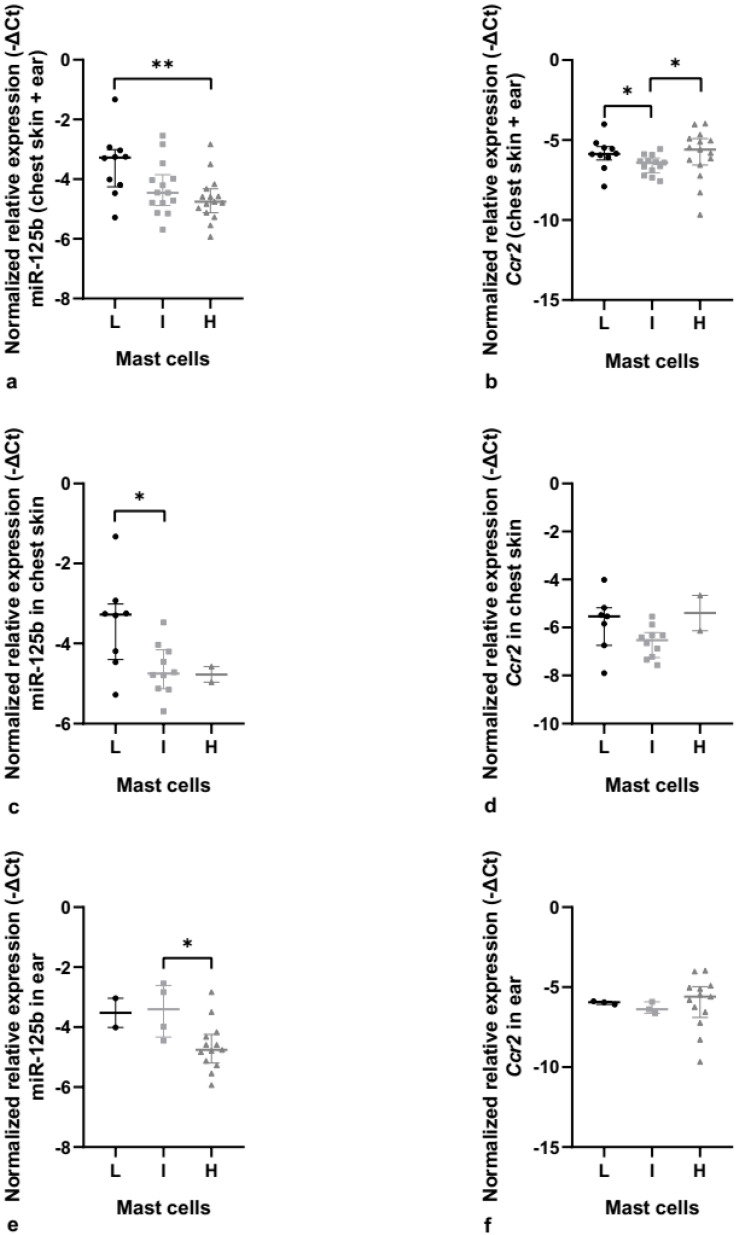
MiR-125b and *Ccr2* relative expression in K14-HPV16 transgenic mice. (**a**) MiR-125b relative expression in chest skin and ear tissue, together. There are statistically significant differences between the low and high mast cell counts (** *p* < 0.01). (**b**) *Ccr2* relative expression in chest skin and ear tissue, together. We observed statistically significant results between the low and intermediate mast cell counts (* *p* < 0.05) and between the intermediate and high mast cell counts (* *p* < 0.05). (**c**) Normalized relative expression of miR-125b in chest kin. Statistically significant differences were observed between the low and intermediate mast cell counts (* *p* < 0.05). (**d**) Normalized relative expression of *Ccr2* in chest skin. (**e**) MiR-125b relative expression in ear tissue. Statistically significant differences were found between the intermediate and high mast cell counts (* *p* < 0.05). (**f**) *Ccr2* relative expression in ear tissue. L: low mast cell count; I: intermediate mast cell count; H: high mast cell count.

Next, we performed the same analysis for miR-125b-5p involving both organs and observed statistically significant results between the low mast cell count and the high mast cell count (*p* = 0.008; 2^−ΔΔCt^ = 2.09) (Figure 7a). When focusing on each organ separately, there was a statistically significant difference between the low mast cell count and the intermediate mast cell count (*p* = 0.026; 2^−ΔΔCt^ = 2.21) in chest skin, and between the intermediate mast cell count and high mast cell count (*p* = 0.031; 2^−ΔΔCt^ = 2.27) in ear tissue, suggesting that this miRNA influences the increased mast cell infiltration in both organs (Figure 6e and Figure 7c). Additionally, we also evaluated *Ccr2* relative expression, since it is one of the essential proteins related to miR-125b. There were statistically significant differences in *Ccr2* expression between the low mast cell count and the intermediate mast cell count (*p* = 0.035; 2^−ΔΔCt^ = 1.61) and between the intermediate mast cell count and the high mast cell count (*p* = 0.036; 2^−ΔΔCt^ = 1.55) when we analyzed both organs together (Figure 7b). However, when analyzing chest skin (Figure 7d) and ear (Figure 7f) separately, no statistical differences were found. 

## 4. Discussion

### 4.1. Mast Cell Infiltration

High-risk HPV is considered one of the main etiological factors associated with the development of cancer [28]. The carcinogenic process associated with a persistent HPV infection develops in several locations through a multistep process, that goes from hyperplastic lesions (alteration in the cell number, shape and size, but still maintaining some differentiation), to dysplastic lesions (with cytological aberrations and loss of tissue differentiation), carcinoma in situ (CIS), and subsequently to invasive squamous cell carcinoma (SCC) [29]. There is a critical connection between the inflammatory microenvironment and cancer [30]. Numerous studies have documented this link in several human carcinomas and the influence of local immunity over patient prognosis and response to therapy [31,32,33,34]. In the early stages of tumor development, immunogenic cancer cells are recognized and eliminated by cytotoxic immune cells such as natural killer (NK) cells and CD8^+^ T cells (process called immunosurveillance) [35]. As the neoplastic tissue evolves into a clinically detectable tumor, there are different inflammatory cells that will impact tumor fate. These so-called tumor-infiltrating immune cells (TIICs) can be trained by tumor cells to favor tumor growth and development. An example of such TIICs are mast cells. Mast cells can secrete a wide range of bioactive molecules (that are contained inside their cytoplasmic granules) that exert both pro- and anti-tumor effects [36]. Several in vivo and in vitro studies showed that mast cells’ pro-tumor activity promotes lymphatic and blood vessel formation, tumor growth and metastasis [37]. 

With regard to the model that we used in this study, the integration of the early region of HPV16 DNA in these transgenic mice allows the spontaneous development of lesions that are very similar to the ones observed in humans, with analogous multistep development [38]. We were able to observe pre-malignant lesions and squamous cell carcinomas on the epidermis of the ear and chest tissue of these transgenic mice [15,38]. Our team and others also used this mouse strain to develop models for tongue base cancer, cervical cancer and penile cancer [39,40,41]. It is thought that a progression of the lesions associated with a persistent HPV infection is accompanied by increased immune cell infiltration, and that was what we wanted to verify whether this happened with mast cells [15,42]. There are previous reports from HPV16-E7 mice showing that accumulation of mast cells in the ear lesions causes local immune suppression [42]. In K14-HPV16 mice, it was shown that cutaneous mast cells release proteases and reorganize stromal architecture and hyperactivate angiogenesis [43]. However, the comparison between cutaneous regions with different susceptibility to HPV-induced carcinogenesis and the mechanisms that promote mast cell accumulation in this mouse model are poorly defined.

The K14-HPV16 mice used in this study had intraepithelial hyperplastic lesions in the chest skin, and dysplastic lesions were restricted to the ear skin [17]. DMBA induced dysplastic lesions in the chest skin and a squamous cell carcinoma in the ear skin [17]. There is evidence that suggests that differences in the histological microenvironment of chest skin and ear tissue of these transgenic mice are correlated with a differential microRNA expression in both of these organs that ultimately affects their susceptibility to cancer [14,15]. 

Our results were consistent with these previous studies, as we identified not only that HPV presence influenced a higher infiltration of mast cells, but also that this accumulation was more prominent in the ear tissue when compared to the chest skin (Figure 1, Figure 2 and Figure 3). Indeed, this increase in mast cells in ear tissue compared to chest skin was observed in both HPV^−^ and HPV^+^ mice. Since DMBA presence did not influence the results, mast cell infiltration seems to only be promoted by HPV presence.

Since our transgenic animals (HPV^+^) had different histological lesions, we were also able to evaluate if the infiltration of mast cells was consistent during the entire carcinogenic process or if different lesions possessed distinct levels of mast cell infiltration. We concluded that as lesions progressed, there was also an increase in mast cell infiltration (Figure 4). This supports the hypothesis that increased mast cell counts are associated with lesion progression and the consequent development of cancer, although additional mechanistic studies are required to establish the tumorigenic contributions of mast cells in this model.

### 4.2. MicroRNAs and Their Putative Roles

MiRNAs possess a dual role, being able to act as tumor suppressors or oncogenes, depending on their target gene functions [44]. Some miRNAs have been described as putative biomarkers for the occurrence and development of HPV-induced cancers [45,46]. Numerous miRNAs have been reported to have a crucial role in HPV-induced carcinogenesis [47,48], including in studies by our group using K14-HPV16 transgenic mice [14,15,49,50]. MiRNAs also modulate many aspects of mast cell behavior (mast cell cycle, proliferation and maturation), being able to act as either inducers and/or suppressors of mast cell biological functions by affecting different targets [51]. Despite the importance of this emerging field of research, the available data on the influence of miRNAs in mast cell function, particularly in cancer, remain very limited.

#### 4.2.1. MiR-223-3p Expression Analysis

MiR-223 in humans is located within the q12 locus of the X chromosome and is mainly expressed by hematopoietic cells [52]. MiR-223 remained highly conserved during evolution, which suggests that it has an important role in several physiological processes, including monocyte-macrophage differentiation, neutrophil recruitment and pro-inflammatory responses [53]. Additionally, miR-223 can also be transferred to non-myeloid cells through lipoproteins or extracellular vesicles [54]. This microRNA’s expression is also deregulated in many types of cancer, from hematological to solid malignancies [55]. Depending on the clinical context, miR-223 can act either as an oncogene (breast cancer) or tumor suppressor (leukemia and hepatocellular carcinoma) [55]. Additionally, studies suggest that downregulation of miR-223 seems to promote mast cell degranulation, while high levels of miR-223 may promote mast cell apoptosis [56,57]. 

In our study, miR-223 was chosen because it was associated with two proteins related to mast cell infiltration and chemotaxis, namely C-X-C motif chemokine ligand 10 (*Cxcl10*) and integrin subunit alpha M (*Itgam*). From miR-223 targets, we chose to study the expression of *Cxcl10,* because from the two the targets of miR-223 it was the one on top of the rank (Table 3). Cxcl10 is a chemokine produced by cancer cells that can activate mast cells and promote their recruitment to solid tumors [58]. Furthermore, this chemokine can stimulate mast cells to secrete diverse soluble factors that support invasion, proliferation and survival of tumor cells [59,60].

Our results suggest that miR-223 may interfere with mast cell infiltration in both chest skin and ear tissue of our transgenic mice, as we observed a tendency toward decreased miR-223 expression while mast cell counts increased. However, this tendency was not statistically significant when analyzing chest skin (Figure 6c) and ear (Figure 6e) separately, possibly due to the lack of statistical power due to the small sample size in some of the groups. Future studies should increase the sample size. Regarding *Cxcl10,* we were able to verify that an increase in *Cxcl10* levels was associated with an increase in mast cell infiltration in chest skin and ear tissue lesions. Thus, we hypothesize that a decrease in miR-223 relative expression may lead to an increase in *Cxcl10*, which will probably increase mast cell infiltration in the tissue.

#### 4.2.2. MiR-125b-5p Expression Analysis

MiR-125b has gained special interest in the field of cancer research because it is found aberrantly expressed in a great variety of tumors [61]. This miRNA is the human orthologue of lin-4, one of the first miRNAs to be identified in *C. elegans* [62]. The function of miR-125b diverges in different cancers depending on the molecular contexts and surrounding tumor microenvironment [61]. It can either act as an oncogene (e.g., nasopharyngeal carcinoma and gastric cancer) or as a tumor suppressor (e.g., breast cancer and colorectal cancer) [63]. Mature miR-125b is generated from two genes (miR-125b-1 and miR-125b-2), both situated in fragile sites commonly deleted in these types of cancer and that imply its loss of function [64].

In general, miR-125b is involved in different cellular processes such as inflammation, cell proliferation and cell cycle regulation. MiR-125b is capable of directly targeting p53, which is known for maintaining genome stability and playing a central role in apoptosis regulation [65]. Furthermore, miR-125b has been shown to interact with multiple mRNAs, including apoptosis regulators such as Bak-1, Bcl-2 and Bcl-w [66,67].

MiR-125b has a central role in productive HPV infection through the regulation of papillomavirus ORF L2 [68]. L2 is associated with the import of HPV DNA to the nucleus, and its inactivation by miR-125b results in loss of viral infectivity [69,70]. Shortly after HPV infection, there is an increased expression of miR-125b that can be explained by interaction with the viral oncoproteins, and that promotes the immune response and inflammation process [71]. As lesions progress, there is a significant decrease in the relative expression of miR-125b [72]. Regarding our bioinformatic analysis, miR-125b was also associated with two proteins related to mast cell infiltration and mast cell chemotaxis, namely C-C motif chemokine receptor 2 (*Ccr2*) and tumor necrosis factor (*Tnf*) (Table 3). 

The results showed a decrease in miR-125b relative expression as the number of mast cells infiltrated in the tumor increased, which we hypothesized could be mediated by increased *Ccr2* expression levels. *Ccr2* is a chemokine that is thought to promote mast cell degranulation and to stimulate an increase in carcinogenesis-associated inflammation mediated by mast cells [73,74]. However, *Ccr2* was not significantly regulated in relation to mast cell infiltration, suggesting that miR-125b may be regulating mast cell infiltration via other downstream effectors. In this context, the expression of *Tnf*, which was also identified as a major regulated protein in our network, should be explored in future studies.

## 5. Conclusions

With this work and its findings, we conclude that HPV16 promotes the progressive accumulation of mast cells in intraepithelial lesions, potentially promoting carcinogenesis. Additionally, we also came to the conclusion that miR-223-3p and miR-125b-5p may be orchestrating pathways that regulate mast cell infiltration in chest skin and ear tissue. There was increased mast cell infiltration in the microenvironment of ear tissue compared with chest skin in both HPV^−^ and HPV^+^ mice. This finding may be a possible explanation for the differences in the severity of the lesions in these two cutaneous sites, observed in HPV^+^ mice.

## Figures and Tables

**Table 1:** Differential mast cell count in different histological lesions of both organs together (**a**), and chest skin (**b**) and ear tissue (**c**) separately. Low, intermediate and high mast cell count values were obtained in the IBM SPSS software platform by ranking the mast cell count in terciles; Low represents a mean mast cell per camp of 0 to 2 mast cells, intermediate refers to a mean of 2 to 9 mast cells per camp, and high represents a mean of 9 to 33 mast cells per camp.

a						
		Histology				Total
		Normal	Hyperplasia	Dysplasia	Carcinoma	
Mast cells Average (Chest skin + Ear)	Low mast cells	11	3	0	0	14
	Intermediate mast cells	3	4	8	0	15
	High mast cells	0	7	7	1	15
	Total	14	14	15	1	44
**b**						
		**Histology**				**Total**
		**Normal**	**Hyperplasia**	**Dysplasia**		
Mast cells Average (Chest skin)	Low mast cells	6	3	0		9
	Intermediate mast cells	0	4	6		10
	High mast cells	0	1	1		2
	Total	6	8	7		21
**c**						
		**Histology**				**Total**
		**Normal**	**Hyperplasia**	**Dysplasia**	**Carcinoma**	
Mast cells Average (Ear)	Low mast cells	5	0	0	0	5
	Intermediate mast cells	3	0	2	0	5
	High mast cells	0	6	6	1	13
	Total	8	6	8	1	23

**Table 2:** Top 10 hub proteins.

Rank	Name	Score	Protein
1	ENSMUSP00000132453	3.03 × 10^27^	CCR2
2	ENSMUSP00000025263	3.03 × 10^27^	IL4
2	ENSMUSP00000000889	3.03 × 10^27^	TNF
4	ENSMUSP00000031322	3.03 × 10^27^	CXCL15
5	ENSMUSP00000031327	3.03 × 10^27^	CXCL1
6	ENSMUSP00000047646	3.03 × 10^27^	CXCL10
7	ENSMUSP00000027061	3.03 × 10^27^	IL13
7	ENSMUSP00000020650	3.03 × 10^27^	IL17A
9	ENSMUSP00000074885	3.03 × 10^27^	CXCL2
10	ENSMUSP00000068468	3.03 × 10^27^	ITGAM

**Table 3:** MicroRNAs found in online databases with respective targets and scores (data last accessed in 10 April 2021). TargetScan 7.2 (https://www.targetscan.org/mmu_72/) relies on conservation of binding sites and divides miRNA families into broadly conserved (conserved across most vertebrates, usually to zebrafish), highly conserved (meaning that they are conserved across most mammals, but usually not beyond placenta) and poorly conserved (all others) [24]. miRDB (http://mirdb.org/) prediction scores are between 50 and 100, assigned by new computational target prediction algorithms (>80 considered most likely to be real) [19]. miRmap (http://mirmap.ezlab.org), ranks potential targets according to repression strength (mediated by RISC), from 0 to 100%, with 100% representing the strongest repression [25]. miRwalk (http://mirwalk.umm.uni-heidelberg.de), incorporates putative targets of 13 prediction datasets, with results being expressed in *p*-values between 0 and 1, representing the probability that a candidate target site is a true target site (high *p*-values are better) [26]. miRTarBase 8.0 (http://miRTarBase.mbc.nctu.edu.tw/)/TarBase v.8 (http://www.microrna.gr/tarbase) are experimentally validated by low throughput techniques (reporter assays, qPCR, Western blot or enzyme linked immunosorbent assays) or high throughput techniques (microarray or proteomics experiments) [23,27]. HITS-CLIP, high-throughput sequencing of RNAs isolated by crosslinking immunoprecipitation; LRA/WB, luciferase reporter assay/Western blot.

MicroRNAs	Target	Databases (Score)
**miR-466l-3p**	IL-4	TargetScan (poorly), miRDB (100), miRmap (93,99)
	TNF	miRDB (100)
	CXCL1	miRDB (95)
	IL-13	miRDB (80)
	IL-17A	TargetScan (poorly), miRDB (96)
	CXCL2	miRDB (98), miRmap (98,64)
**miR-466k**	CXCL15	miRTarBase (HITS-CLIP), miRDB (100), miRmap (99,98), miRwalk (0.92)
	ITGAM	TargetScan (poorly), miRDB (100), miRmap (99,95), miRwalk (0.87)
**miR-223-3p**	CXCL10	miRDB (90), miRmap (94,46), TarBase (-) *
	ITGAM	miRwalk (1), TarBase (-) *
**miR-125b-5p**	TNF	miRTarBase (LRA/WB)
	CCR2	miRwalk (1), Tarbase (-) *

* Present in the database but with no score associated.

**Table 4:** Kruskal–Wallis test for the normalized relative expression of the four microRNAs along mast cell count.

Normalized Relative Expression (−ΔCt)	*p* Value
miR-466l-3p	0.408
miR-466k	0.999
miR-223-3p	0.035
miR-125b-5p	0.024

## Data Availability

The data presented in this study are available on request from the corresponding author.

## Supplementary Materials

The following supporting information can be downloaded at: https://www.mdpi.com/article/10.3390/cancers14092216/s1, Figure S1: Histological analysis of lymph node samples, DAB-hematoxylin. (**a**). Lymph node with mast cell infiltration observed in mice, 100×. (**b**). Same lymph node from figure (**a**). with high magnification 500×. (**c**). Lymph node without primary antibody, 100×. (**d**). Same lymph node from figure c. with high magnification 500×.
