# Supplementary material for: Characterizing the Inflammatory Microenvironment in K14-HPV16 Transgenic Mice: Mast Cell Infiltration and MicroRNA Expression
Source: Cancers (Basel). 2022 Apr 28;14(9):2216. doi: 10.3390/cancers14092216 (PMC9099850; doi:10.3390/cancers14092216)
Supplement: Supplementary file 1 [file cancers-14-02216-s001.zip › cancers-1664805-supplementary.pdf]

Supplementary Materials

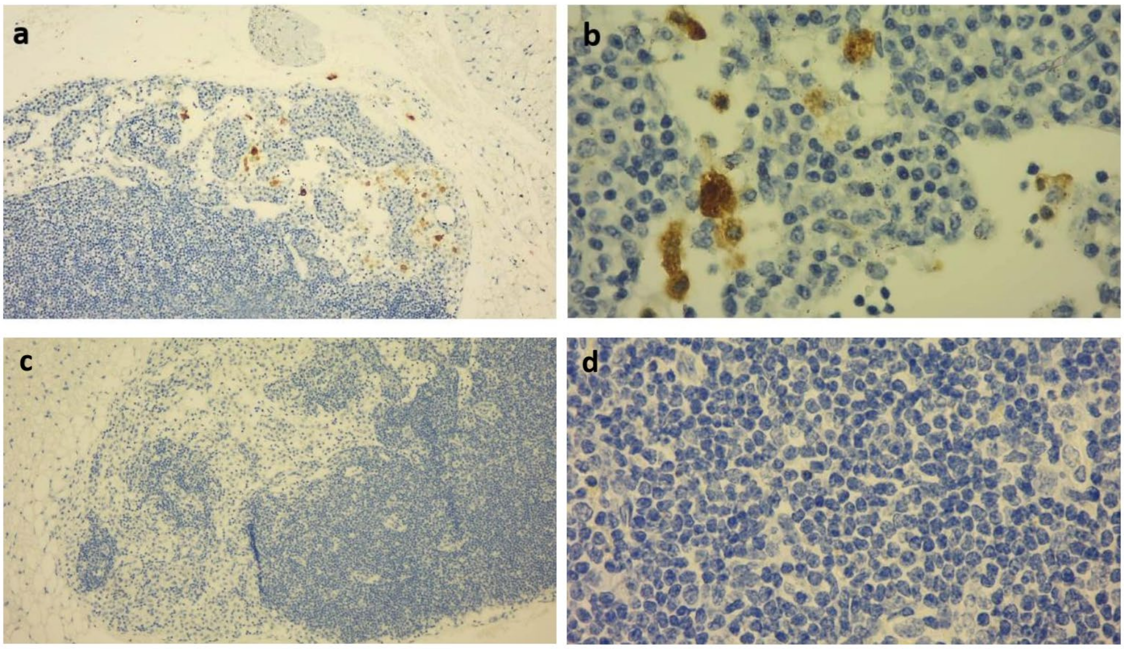

Figure S1. Histological analysis of lymph node samples, DAB-hematoxylin. (**a**). Lymph node with mast cell infiltration observed in mice, 100×. (**b**). Same lymph node from figure a. with high magnification 500×. (**c**). Lymph node without primary antibody, 100×. (**d**). Same lymph node from figure c. with high magnification 500×.
